# Cytomegalovirus in children undergoing haematopoietic stem cell transplantation: a diagnostic and therapeutic approach to antiviral resistance

**DOI:** 10.3389/fped.2023.1180392

**Published:** 2023-05-30

**Authors:** Jocelyn Hume, Emma L. Sweeney, Kym Lowry, Chris Fraser, Julia E. Clark, David M. Whiley, Adam D. Irwin

**Affiliations:** ^1^ The University of Queensland Centre for Clinical Research (UQCCR), Faculty of Medicine, The University of Queensland, Brisbane, QLD, Australia; ^2^ Central Microbiology, Pathology Queensland, Brisbane, QLD, Australia; ^3^ Blood and Bone Marrow Transplant Program, Queensland Children’s Hospital, Brisbane, QLD, Australia; ^4^ Infection Management and Prevention Service, Queensland Children’s Hospital, Brisbane, QLD, Australia

**Keywords:** haematopoietic stem cell transplant, cytomegalovirus, antiviral resistance, pediatric, emerging therapies

## Abstract

Cytomegalovirus (CMV) is a ubiquitous virus which causes a mild illness in healthy individuals. In immunocompromised individuals, such as children receiving haematopoietic stem cell transplantation, CMV can reactivate, causing serious disease and increasing the risk of death. CMV can be effectively treated with antiviral drugs, but antiviral resistance is an increasingly common complication. Available therapies are associated with adverse effects such as bone marrow suppression and renal impairment, making the choice of appropriate treatment challenging. New agents are emerging and require evaluation in children to establish their role. This review will discuss established and emerging diagnostic tools and treatment options for CMV, including antiviral resistant CMV, in children undergoing haematopoietic stem cell transplant.

## 1. Introduction

Cytomegalovirus (CMV) is a double stranded, linear DNA virus that is approximately 236 kilobase pairs (kbp) in length and belongs to the *Herpesviridae* family. The *Herpesviridae* family is divided into three sub-families, based on their biological properties: alpha herpesviruses (including the causative agents of “cold sores” and “genital herpes”, Herpes Simplex virus), beta herpesviruses (including CMV) and gamma herpesviruses (including Epstein Barr virus).

CMV is a ubiquitous herpesvirus that results in mild, self-limiting infections in healthy individuals. The seroprevalence in Australia is estimated to be over 30% in infants, increasing to over 50% by adulthood, and is higher in lower socio-economic areas (1, 2). As with other herpesvirus infections, CMV remains latent after primary infection, for the life of the host. This latent CMV can reactivate during periods of stress or when the host immune system becomes compromised, such as during solid organ transplantation (SOT) or haematopoietic stem cell transplantation (HSCT). In HSCT, CMV disease including pneumonitis, hepatitis, colitis and retinitis occurs in approximately 50% of patients with CMV reactivation, without antiviral intervention. Reactivation is associated with an approximately 3-fold increase in non-relapse mortality in HSCT recipients (3–5). In this setting, CMV reactivation may require prolonged antiviral treatment. This may select sub-populations of virus with reduced antiviral susceptibility, leading to CMV antiviral resistance and treatment failure (6). Current testing for antiviral resistance in CMV is difficult to access, slow and expensive, limiting its diagnostic value.

## 2. Cytomegalovirus in haematopoietic stem cell transplantation

In HSCT, donor progenitors of the immune system are transplanted into patients as definitive treatment for a range of haematological, immune, metabolic, and other disorders. The indications for HSCT in children have grown dramatically over the last four decades (7). Immunosuppressive drugs, graft failure, and Graft vs. Host Disease (GvHD) all result in significant immune suppression and play a role in the increased risk of morbidity and mortality in children receiving HSCT (8–10). Paediatric HSCT recipients require extended CMV monitoring after GvHD due to increased risk of developing CMV reactivation long after transplantation (11, 12). Infectious complications are common and the risk of infection increases with the intensity and duration of immunosuppressive therapy (9, 13).

Children undergoing HSCT may experience either primary infection with CMV or reactivation of a latent infection. Recent studies estimate that CMV reactivation occurs in approximately 20% of HSCT recipients (14, 15). It commonly presents asymptomatically and occurs an average of 8-weeks post-transplantation (8, 16, 17). The risk of CMV reactivation in patients is associated with the serostatus of the patient (recipient) and the transplant donor. Unlike in solid organ transplantation, seropositive HSCT recipients are at highest risk of reactivation owing to the prolonged absence of T-cell mediated immunity to control CMV replication (10, 13, 18).

## 3. The diagnostic approach to CMV in HSCT

Prevention of CMV disease may be achieved by a prophylactic or pre-emptive approach. Antiviral prophylaxis in HSCT is complicated by drug-drug interactions and toxicity, so it is common practice to pursue a pre-emptive approach in which regular surveillance of blood for CMV reactivation initiates pre-emptive therapy. Pre-emptive therapy is the most-used approach in Australasian paediatric HSCT recipients (19, 20). The 2017 European Conference on Infections in Leukaemia (ECIL7) recommend weekly sampling until at least 100 days post-transplant; however, sampling frequency and duration may be increased in patients deemed to be at high risk of infection (21, 22). In some cases, CMV reactivation or primary infections may occur more than 10 years post transplantation. These late reactivations typically have worse outcomes, likely due to delayed diagnosis (23–25).

### 3.1. Polymerase chain reaction

PCR is the most common method used in diagnostic laboratories for CMV detection and can be qualitative or quantitative (qPCR). PCR assays target and amplify conserved sections of the CMV genome with the inclusion of fluorescent dyes or probes used to assess the presence of the target within a patient's sample. qPCR has superseded testing for CMV antigenemia, and may be performed on various specimen types, including blood, urine, bronchoalveolar lavage and organ tissues (26). Surveillance as part of a pre-emptive treatment approach is performed on blood.

Interpretation of qPCR results are complicated by a lack of standardised diagnostic methods, variable specimen types, and differences in collection schedules and reference standards. In 2010, the World Health Organisation (WHO) established an international standard to be used for qPCR to ensure the reproducibility of results and to reduce variation in reporting between laboratories (27, 28). Nonetheless, there is no consensus for the viral copy number at which treatment should be initiated (21).

The monitoring of CMV viral loads can help to infer the emergence of antiviral resistance. Consensus recommendations define refractory infection when the viral load increases by more than 1 log_10_ after at least 2 weeks of appropriate antiviral therapy. In the absence of cell-mediated immunity following HSCT, viral loads may take several weeks to reduce despite effective treatment. However, it would be reasonable in this setting to initiate testing for antiviral resistance (21).

## 4. Antiviral resistance in CMV

Antiviral resistance in CMV occurs due to single nucleotide mutations in genes targeted by CMV antiviral drugs, such as DNA polymerase (*UL54*), terminase subunit (*UL56*, *UL51* & *UL89*) and viral protein kinase (*UL97*). The genes and specific single nucleotide polymorphisms (SNPs) associated with CMV antiviral resistance are summarised in Table 1. Mutations in the viral protein kinase (*UL97*) are the most common mutations reported and reduce the phosphorylation of Ganciclovir, preventing Ganciclovir from being added to the growing DNA chain and causing it to be ineffective (29). Mutations in *UL97* may also prevent its binding of Maribavir, causing Maribavir to become ineffective (30). Mutations in the viral DNA polymerase (*UL54*) can cause resistance to Ganciclovir, Cidofovir and Foscarnet. Some mutations cause increased exonuclease activity of the viral DNA polymerase, which allows the cleavage of areas containing Ganciclovir or Cidofovir thereby reducing their effectiveness (31). Structural changes in the viral DNA polymerase prevent its binding to Foscarnet, causing Foscarnet to become ineffective (32). Mutations in any of the genes that make up the viral terminase subunit (*UL56*, *UL51* and *UL89*) can prevent its binding to Letermovir, allowing it to continue cleaving the concatemers and hence causing Letermovir to become ineffective (33).

**Table 1:** List of mutations and deletions causing CMV antiviral resistance, ganciclovir [(GCV) including valganciclovir], cidofovir (CDV), foscarnet (FOS) and maribavir (MBV) (32, 33, 49, 85, 115–124).

Drug	Gene	Mutations/deletions	Drugs	Gene	Mutations/deletions
MBV	***UL27***	L337M*, V353A*, L397R*, T409M, H411Y/L*/N, H411N	**GCV & MBV**	***UL97***	F342Y/S*, V356G*, D456N*, V466G, C480F/R*, V466G, P521L, Y617del*
	***UL97***	L348V			
GCV	***UL97***	F342Y, K359E/Q/N, E362D, L405P, M460V/I/T/L, V466G, A505T, C518Y, H520Q, P521L, A591V, C592G, A594V/T/E/G/P/S, 591–594del, 591–607del, 595–603del, 595del, 600del, 601–603del, 601del, I610T, A613V, L595S/F/W, E596G/Y, G598S, K599T, C603W/R/S, C607Y/F, I610T	**GCV & CDV**	***UL54***	D301N, E303D*/G*, N408D/K/S, N410K, F412C/l/S/V*, D413E/A/N/Y*, K488R*, K500N, L501I/F, T503I, A505V, K513E/N/R/T, D515Y, L516P/W/R*, I521T, P522A/S, C524del, V526L, C539G/R*, A543P*, L545S/W, I726T/V, V823A, A987G
	***UL54***	524del, P829S*, L957F	**GCV & FOS**	***UL54***	S290R*, Q578L, D588N, L776M, V781I, V787A/L, L802M, A809V, T821I, M844V*, E951D*, E989D
FOS	***UL54***	N495K, T552N*, L565V, S585A*, D588E, F595I*, T700A, V715A/M, E756D/Q/G, Q783R*, V798A*, T813S, T838A, M844T*, V946l*, A987V	**GCV & FOS & CDV**	***UL54***	K493N, Q578H, D588N, Q589H, H600L, E756K, L773V, V787E, V812l, T813S, A834P, G841A/S, A928T, 981–982del
CDV	***UL54***	P497S, D542E*, A543V, K805Q			
Letermovir					
Gene		***UL56***		***UL51***	***UL89***
Mutations/Deletions		M3I/V*, C25F, L26P*, F41L*, I48M*, L51M, A103V*, E141STOP*, N148D*, E157G*, Q182K*, Q213R*, S229F, V231A/L, N232Y, Q234R, V236A/L/M, E237D/G, V236M, L241P, L243P, T244K/R, L254F, S255L*, L257F/I, K258E, F261C/l/S, S262C, S269G*, E276G*, I313V*, Y321C, C325F/Y/R/W, L328V/I, M329T, H335Y, E339G, K350R, V363I, A365S, N368D/I, R369G/S/T/M/S/K, S378N*, T399I, S445N, S445-S447deL*, E485G*, I535V*, E542deL*, Y575C*, M641T*, L658S*, Y667H*, S705F*, V706A*, L750P*, Y757H*, Y775I*, R816W*, P846l*		P91S, V113L	K41E*, N74S*, S102F*, F124L*, T132A*, V146I*, P176S*, H243R*, H246R*, D309G*, N320H, L323P*, T331A*, D344E, T350M, M359I, S373G*, M406V*, N426D*, L458P*, S521G*, L522P*, A532T*, I572V*, Q625STOP*, T637A*, V656A*

*Suspected resistance.

Mutations in the CMV genome may arise under selective pressure, such as prolonged or subtherapeutic antiviral therapy (10, 34). Mixed infections in which different variants of CMV, exhibiting different susceptibility profiles occur. These are of particular concern in HSCT recipients who may experience prolonged periods of CMV reactivation despite prolonged antiviral therapy (26, 35–37).

The prevalence of Ganciclovir resistant CMV infections in HSCT recipients has been reported up to 9.4% (25, 38–42). Due to the limitations of resistance testing these rates may be under-estimated (40, 41, 43). Antiviral resistance is often associated with multiple resistance mutations. An Australian study found rates of infections in solid organ transplant (SOT) recipients harbouring both *UL54* and *UL97* mutations had increased over a six-year period (26). There is little information regarding mortality in antiviral resistant CMV infections. One study reported mortality to be 11% higher in SOT recipients with Ganciclovir resistant CMV infections, while two other studies reported mortality of 25% and 69% respectively (6, 39, 44).

Although Letermovir is a relatively new drug, there are reports of CMV resistance to Letermovir developing rapidly (45, 46). Maribavir may be useful in treating Ganciclovir resistant CMV infections (47, 48). Cross resistance to Maribavir and Ganciclovir arises rarely despite their dissimilar mechanisms of action (49). Mutations in *UL27* gene have been associated with reduced effectiveness of Maribavir, however, this could be a result of counteractivity for the loss of the viral protein kinase, rather than causing Maribavir resistance (49).

## 5. CMV antiviral resistance testing

Sanger sequencing is the most commonly used method to identify mutations in *UL97* and *UL54* known to be associated with resistance to ganciclovir and foscarnet (26, 34, 37, 50). Sanger sequencing requires viral loads of at least 1,000 IU/ml for successful characterisation, and is unable to differentiate variants that make up less than 20%–30% of the overall viral population. As a consequence this method may fail to identify mixed infections, or low-levels of resistance in a predominantly susceptible population (35, 51). Patients should be tested for antiviral resistance whenever resistance is suspected, even after mutations have been identified, as additional resistance mutations can develop (26). The requirement of high viral loads, alongside the extended turnaround times can hinder the desirability of sequencing for antiviral resistance testing. Next generation sequencing (NGS) technologies are a higher resolution tool capable of detecting minority variants down to as little as 1%, and may be performed in samples with a lower viral load (52–54). Currently, due to the cost per sample, lack of availability of NGS within all pathology laboratories, specialised skills required for analysis, and scarcity of databases which summarise the clinically relevant antiviral resistance mutations for use in a bioinformatics pipeline, NGS assays are not widely used for detection of CMV antiviral resistance.

## 6. Novel diagnostic approaches

### 6.1. Interferon gamma release assay for CMV (IGRA-CMV)

A recently commercialised diagnostic assay is the IGRA-CMV which measures interferon-*γ* that is released by the patients' T-lymphocytes when exposed to CMV antigens *ex vivo* (55, 56). These assays indicate immunity to CMV and correlate with the subsequent risk of progression to CMV reactivation or disease. The IGRA-CMV has been evaluated in HSCT recipients and has demonstrated the potential to assist clinicians when to start and stop antiviral treatments (55–57). The ECIL7 supports the use of IGRA in addition to qPCR to support risk stratification and guide the treatment of HSCT recipients with CMV infections, but there are currently no clinically validated thresholds for intervention (21).

### 6.2. Digital PCR

Digital PCR offers advantages over conventional PCR methods by quantifying viral loads without the need for a calibration curve. It exhibits improved precision without a loss of sensitivity (58). It is also possible to quantify CMV antiviral resistant mutations, however this method requires unique assays for every mutation, in addition to specialised instrumentation (59). The role of digital PCR for CMV monitoring is currently limited by the workflow advantages of conventional PCR methods.

### 6.3. Viable and non-viable CMV

The COVID-19 pandemic has sparked discussions around viable and non-viable virus in patients with detectable SARS-CoV-2 long after clinical symptoms have ceased (60, 61). Persistent viraemia despite appropriate treatment complicates treatment decisions. Differentiating between viable and non-viable CMV could allow for early detection of drug failure and may also provide a means of assessing drug resistance without relying on detection of specific resistance mutations. To date no such commercial systems exist for CMV.

## 7. Established therapies

### 7.1. Ganciclovir

Ganciclovir is a first-line antiviral drug used for treating CMV (21). It acts as a guanosine analogue that is initially phosphorylated by viral protein kinase (*UL97*) (62–64). The phosphorylated form competes with guanosine triphosphate by incorporating itself into the growing DNA chain, stalling the DNA polymerase (*UL54*) and preventing further transcription of the viral DNA (64). Due to the requirement of CMV viral protein kinase activity for initial phosphorylation, Ganciclovir may have reduced efficacy at low CMV viral loads (65).

Importantly, Ganciclovir can also disrupt transcription of human cellular DNA, hence it can be toxic to the patient (66, 67). Ganciclovir is given intravenously, requiring patients to attend hospitals to receive treatment and often requiring central venous lines. Twenty years after the development of Ganciclovir, its prodrug, Valganciclovir was approved for use in 2001. Valganciclovir has the same mode of action and efficacy as Ganciclovir (68), however it can be taken orally and has a favourable toxicity profile (8). Ganciclovir may still be required in severe CMV infections and where oral absorption cannot be assumed (26). Effective ganciclovir exposures may be assessed by therapeutic drug monitoring (TDM), though this remains uncommon (69).

There is controversy surrounding appropriate dosing of Ganciclovir in paediatric patients. Institutional guidelines vary in their use of weight or body surface area-based dosing and there are few data to support a pharmacodynamic target. Ganciclovir is known to cause bone marrow suppression, and may cause higher rates of hepatotoxicity in children compared with adults, making appropriate dosing a priority (70). Though weight-based dosing achieved lower ganciclovir exposures than body-surface area based dosing in paediatric SOT recipients, these exposures achieved a virological clearance (71).

### 7.2. Foscarnet

Foscarnet binds to the viral DNA polymerase (*UL54*), occupying the position where phosphates of incoming deoxynucleotide triphosphates (dNTPs) would sit, hence preventing normal pyrophosphate release and halting the viral DNA polymerase (72). Foscarnet must be given intravenously, and is associated with significant renal toxicity (34). It has fewer effects on the bone marrow and as such, may be used as first-line therapy in the immediate post-transplant period. It is otherwise used as a second-line therapy when there are adverse events associated with ganciclovir or there is identified or suspected resistance to Ganciclovir (21, 34). Renal dysfunction caused by Foscarnet can persist in patients for up to 6 months after the discontinuation of the drug (34). Paediatric patients experience higher rates of Foscarnet related thrombocytopenia compared with adults (70). One study used a combination of Ganciclovir and Foscarnet to treat high-risk paediatric HSCT recipients and found alternating drugs daily was very effective in preventing the development of CMV disease, however 30% of patients discontinued treatment due to toxicity (73).

### 7.3. Cidofovir

Cidofovir is a cytosine analogue that is phosphorylated by cellular enzymes and incorporated into DNA to slow elongation (74). Cidofovir has a long intracellular half-life, which is advantageous as it is not orally available and must be administered intravenously (75). An orally available lipid prodrug of Cidofovir (Brincidofovir) failed to reduce CMV infection in a randomised controlled-trial of prophylaxis in adults undergoing HSCT, and caused significant gastro-intestinal toxicity which limited its use (76).

The half-life of Cidofovir is longer in paediatric patients, however it is similarly tolerated to adults (75). Cidofovir has limited effectiveness in treating Ganciclovir-resistant infections and is associated with significant ocular and renal toxicity in paediatric patients (77).

## 8. Emerging therapies

### 8.1. Letermovir

Letermovir was approved by the FDA in 2017 for prophylaxis in adult HSCT recipients at high risk of CMV reactivation (78). The CMV genome is circularised during replication, allowing it to replicate rapidly in both directions, the product of which is a very long strand of DNA of multiple sets of CMV genomes linked end to end, known as concatemers (79). These concatemers are normally cleaved and packaged into individual virions by the viral terminase subunit (*UL51*, *UL56* & *UL89*); however, Letermovir binds to the viral terminase subunit, preventing this mechanism and leaving the long viral DNA incapacitated (80, 81).

Letermovir specifically targets CMV and has no effect on other herpesviruses (82), and can be administered both intravenously and orally, with side-effects reported to be uncommon (83). Patients receiving cyclosporin or tacrolimus (used for immunosuppression in transplant recipients) in addition to Letermovir must be monitored closely, as drug-drug interactions can lead to increased absorption of cyclosporin (84). As with the other drugs, there is evidence of resistance developing after treatment (85).

Letermovir significantly reduced the number of CMV infections compared to placebo in a Phase 3 randomised controlled trial of prophylaxis in adults, and was well tolerated (86). It has become the drug of choice for prophylaxis in centres in Germany, Italy, Japan, and the USA (87–90). Data are emerging to support the use of letermovir to prevent CMV in children following HSCT (91, 92), while it has also been used successfully as pre-emptive therapy (93, 94). A Phase 2 study of Letermovir prophylaxis in children undergoing HSCT is ongoing (ClinicalTrials.gov identifier NCT03940586) (95).

### 8.2. Maribavir

Maribavir is orally available and functions by competing with adenosine triphosphate (ATP) to bind to the viral protein kinase (48). When bound to Maribavir, the viral protein kinase is unable to phosphorylate nucleotides, resulting in halted transcription of the viral DNA (48). Maribavir was approved by the FDA in 2021 for treating CMV infections that were not responding to conventional therapies (96). Considering Maribavir prevents the viral protein kinase from phosphorylating nucleotides, it also prevents phosphorylation of Ganciclovir, causing it to become ineffective, hence Maribavir and Ganciclovir cannot be administered together (97). Maribavir is ineffective at preventing CMV when used as a prophylactic treatment, which may be explained by the non-essential nature of the viral protein kinase (CMV can recruit host cell kinases to continue viral replication) (98). Resistance may also develop rapidly, with one study reporting resistance developing at an average of 16 weeks after starting Maribavir therapy (52). To the extent of our knowledge, there have been no studies examining the tolerability of Maribavir in paediatric patients.

### 8.3. CMV-specific cytotoxic T cells (CMV-CTLs)

There has been substantial interest in the adoptive transfer of donor-derived CMV-CTLs to treat refractory CMV infections following HSCT since its first demonstration (99). The use of CMV-CTLs appears to accelerate CMV-specific immune reconstitution and reduce viral load (100), and is particularly attractive in the context of refractory or resistant infection (101). The techniques required to obtain CMV-CTLs are time-consuming and costly, so far limiting their widespread evaluation, and adoption into clinical practice. Despite this, their promise is reflected by inclusion in international guidelines such as ECIL7 which support their use for pre-emptive therapy in high-risk patients, and for the treatment of antiviral resistant infections (21).

Posoleucel is a third-party multi-virus T cell therapy which has been evaluated in children and adults undergoing HSCT. In an open-label Phase 2 study of 58 patients (including 18 children) there was a complete response in 11/24 patients and partial improvement in 12/24 patients with CMV infections suspected to be antiviral resistant. Posoleucel was effective in 10/12 patients with two or more viral infections. It was well tolerated with no evidence of an increased risk of GvHD (102). Larger studies are required to prove efficacy of this therapy and demonstrate the risk of GvHD, in addition to how the drug is tolerated in paediatric HSCT recipients.

### 8.4. CMV vaccines

There is presently no licensed vaccine to prevent CMV despite numerous candidates being evaluated in clinical trials (103). An mRNA vaccine candidate supported by Moderna TX, Inc (104). is currently undergoing a phase 3 trial in women of childbearing age, with the aim of preventing congenital CMV. It is expected to complete in 2025 (ClinicalTrials.gov identifier NCT05085366) (105). Also under development is a CMV bivalent subunit vaccine, which has demonstrated promising immune responses in mice, with the hopes to begin human trials in the coming years (106). The availability of a vaccine may not improve care for immunocompromised patients due to their inability to mount a suitable immune response.

## 9. Off label treatment options

### 9.1. Leflunomide

Leflunomide is an immunosuppressant used in rheumatoid arthritis. It has shown effectiveness as an off-label treatment for CMV infections by interfering with virion assembly (107). Successful treatment of Ganciclovir resistant infections has been reported with a combination of Foscarnet and Leflunomide (108). However, Leflunomide has displayed reduced effectiveness in the presence of higher CMV loads (109, 110). Despite Leflunomide being well tolerated for treating rheumatoid arthritis, higher doses are required to treat CMV, causing side-effects, such as diarrhoea, anaemia, and increased liver function tests (109). ECIL7 recommends Leflunomide is only used to treat antiviral resistant CMV infections and should be used in conjunction with antiviral therapy (21, 22).

### 9.2. Artesunate

Artesunate is an antimalarial drug that has been used off-label for the treatment of CMV infections (111, 112). Artesunate binds to a human cellular protein called vimentin; vimentin is normally cleaved during CMV infection, suggesting it has a role in controlling CMV. Artesunate binding prevents the cleavage of vimentin, in turn inhibiting CMV infection (113). Effectiveness of Artesunate in combination with conventional drugs has been demonstrated in cell culture (114). Clinical studies have reported mixed efficacy in controlling CMV infection in HSCT recipients (112), and its use is only recommended when resistance has developed to conventional drugs (21, 22).

## 10. Summary

CMV is effectively treated using conventional (Ganciclovir and Valganciclovir, Foscarnet and Cidofovir) and emerging antiviral drugs (Maribavir and Letermovir). There is also an immunological approach to treatment (CMV-CTLs) and off-label drugs that have shown some effectiveness in treating refractory CMV infections (Leflunomide and Artesunate). Antiviral resistance in CMV is a growing problem in children undergoing HSCT and there remain significant limitations in our diagnostic approach to resistance. NGS may guide a more nuanced approach to the treatment of mixed infections.
